# Age-related Disparities in Pan-Cancer Mortality and Causes of Death: Analysis of Surveillance, Epidemiology, and End Results (SEER) Data

**DOI:** 10.7150/jca.91758

**Published:** 2024-01-21

**Authors:** Yile Lin, Huiyang Li, Haixiao Wu, Shu Li, Maxim A. Abakumov, Vladimir P. Chekhonin, Karl Peltzer, Kirellos Said Abbas, Alexander D. Makatsariya, Zheng Liu, Jin Zhang, Yuan Xue, Chao Zhang

**Affiliations:** 1 Department of Orthopaedic Surgery, Tianjin Medical University General Hospital, Tianjin, China.; 2 The Sino-Russian Joint Research Center for Bone Metastasis in Malignant Tumor, Tianjin, China.; 3 Department of Gynecology and Obstetrics, Tianjin Medical University General Hospital, Tianjin, China; Tianjin Key Laboratory of Female Reproductive Health and Eugenics, Tianjin Medical University General Hospital, Tianjin, China.; 4 Tianjin Medical University Cancer Institute and Hospital, National Clinical Research Center for Cancer, Key Laboratory of Cancer Prevention and Therapy, Tianjin's Clinical Research Center for Cancer, Tianjin, China.; 5 Department of Public Service Management, School of Management, Tianjin University of Traditional Chinese Medicine, Tianjin, China.; 6 National University of Science and Technology (MISIS), Moscow, Russia.; 7 Department of Medical Nanobiotechnology, N.I Pirogov Russian National Research Medical University, Moscow, Russia.; 8 Department of Research & Innovation, University of Limpopo, Sovenga, Limpopo, South Africa.; 9 Faculty of Medicine, Alexandria University, Alexandria, Egypt.; 10 Department of Obstetrics, Gynecology and Perinatal Medicine, Filatov Clinical Institute of Children's Health, Sechenov University, Moscow, Russia.; 11 Department of Orthopaedic Surgery, The Seventh Affiliated Hospital, Sun Yat-sen University, Shenzhen, China.

**Keywords:** Age, Cause of Death, SEER Program, Standardized Mortality Rate, Trend

## Abstract

Comprehensive analysis of mortality and causes of death (COD) in cancers was of importance to conduct intervention strategies. The current study aimed to investigate the mortality rate and COD among cancers, and to explore the disparities between age. Initially, cancer patients diagnosed between 2010 and 2019 from the surveillance, epidemiology, and end results (SEER) database were extracted. Then, frequencies and percentage of deaths, and mortality rate in different age groups were calculated. Meanwhile, age distribution of different COD across tumor types was illustrated while the standardized mortality ratios (SMR) stratified by age were calculated and visualized. A total of 2,670,403 death records were included and digestive system cancer (688,953 death cases) was the most common primary cancer type. The mortality rate increased by 5.6% annually in total death, 4.0% in cancer-specific death and 10.9% in non-cancer cause. As for cancer-specific death, the age distribution varied among different primary tumor types due to prone age and prognosis of cancer. The top five non-cancer causes in patients older than 50 were cardiovascular and cerebrovascular disease, other causes, COPD and associated conditions, diabetes as well as Alzheimer. The SMRs of these causes were higher among younger patients and gradually dropped in older age groups. Mortality and COD of cancer patients were heterogeneous in age group due to primary tumor types, prone age and prognosis of cancer. Our study conducted that non-cancer COD was a critical part in clinical practice as well as cancer-specific death. Individualized treatment and clinical intervention should be made after fully considering of the risk factor for death in different diagnosis ages and tumor types.

## Introduction

Cancer is a major public health problem and one of the leading of death around the world. According to the latest updated cancer statistics, there were an estimated 1.9 million diagnosed new cancer cases and more than 600,000 deaths from cancer in the United States in 2022^1^. Cancer mortality has declined recently at a slowly accelerated pace. As literature showed, estimated cancer cases in the United States by the year 2040 would be 1.88 million^2^. The landscape of cancer incidence and deaths changed over time. Shifting trend in mortality was an important reference to guide health policy efforts and future research funding allocation.

Due to the continuous progress and development in oncology, cancer-specific survival had been improved correspondingly. Causes of death (COD) was defined as the disease or condition responsible for initiating the chain of events leading to death^3^. Among individuals diagnosed with cancer, cancer was not the only COD in the end. The risk of dying from cancer and non-cancer causes were influenced by primary cancer types, clinical stage, treatment, and age especially. Individuals diagnosed with certain cancer types, such as lung and bronchus cancer, prostate cancer^4^, breast cancer, kidney, and renal pelvis cancer, and liver cancer, were proved to be prone to die of non-cancer causes^5^, ^6^. One of the most common non-cancer COD was reported to be cardiovascular and cerebrovascular diseases^6^, ^7^. With regard to the increasing survivorship from cancer, the identification of cancer patients who were with high risk of dying was warranted. During the process, age played a critical role in cancer mortality and COD, which should not be ignored.

The purpose of the current study was to characterize the mortality and COD among cancer patients diagnosed with single site primary cancer based on the SEER cancer database. Furthermore, age-related disparities were explored. These findings provided an overview of cancer-specific death and non-cancer COD in different age groups among different primary cancer types. Discriminating patients at different age who were at risks to die of various cancers or concurrent non-cancer causes was essential to assist physicians in redistributing cancer care and helping patients profit from the individualized screening strategies.

## Materials and Methods

### Study population selection

Patients diagnosed with malignant cancer were initially extracted and then those died of any cause (cancer-specific death or non-cancer causes) from 2010 to 2019 were included for further analyses. *'One primary only'* was selected in *'Multiple Primary Fields. Sequence number'* option to exclude patients with multiple malignant cancers (≥2 cancers). Patients with unknown age information or with unknown cause of death (COD) were excluded. According to '*Age recode with <1 year old*' variable in SEER program, patients were divided into several groups: 00 years, 01-04 years, 05-09 years, 10-14 years, 15-19 years, 20-24 years, 25-29 years, 30-34 years, 35-39 years, 40-44 years, 45-49 years, 50-54 years, 55-59 years, 60-64 years, 65-69 years, 70-74 years, 75-79 years, 80-84 years and 85+ years.

### Data sources

**Frequencies of deaths and percent weight of primary tumors:** Surveillance, Epidemiology, and End Results (SEER) Program (www.seer.cancer.gov) SEER*Stat Database: Incidence - SEER Research Plus Data, 17 Registries, Nov 2021 Sub (2000-2019) - Linked to County Attributes - Total U.S., 1969-2020 Counties, National Cancer Institute, DCCPS, Surveillance Research Program, released April 2022, based on the November 2021 submission.

**Estimate age-adjusted mortality rate:** Surveillance, Epidemiology, and End Results (SEER) Program (www.seer.cancer.gov) SEER*Stat Database: Incidence-Based Mortality - SEER Research Plus Data, 17 Registries, Nov 2021 Sub (2000-2019) - Linked to County Attributes - Total U.S., 1969-2020 Counties, National Cancer Institute, DCCPS, Surveillance Research Program, released April 2022, based on the November 2021 submission.

**Age distribution in different primary tumors:** Surveillance, Epidemiology, and End Results (SEER) Program (www.seer.cancer.gov) SEER*Stat Database: Incidence - SEER Research Plus Data, 17 Registries, Nov 2021 Sub (2000-2019) - Linked to County Attributes - Time Dependent (1990-2019) Income/Rurality, 1969-2020 Counties, National Cancer Institute, DCCPS, Surveillance Research Program, released April 2022, based on the November 2021 submission.

**Calculate standardized mortality ratios (SMRs):** Surveillance, Epidemiology, and End Results (SEER) Program (www.seer.cancer.gov) SEER*Stat Database: Incidence - SEER Research Plus Data, 17 Registries (excl AK), Nov 2021 Sub (2000-2019) for SMRs - Linked to County Attributes - Total U.S., 1969-2020 Counties, National Cancer Institute, DCCPS, Surveillance Research Program, released April 2022, based on the November 2021 submission.

### Primary tumors and cause of death data

In the present study, we conducted a pan-cancer analysis among eighteen different cancer types. The classification of cancer types was defined according to the variable of 'Site recode ICD-O-3/WHO 2008', which was described in our previous article^5^. The mortality trend and COD among pan-cancer were analyzed and visualized. Groups were set as following: oral cavity and pharynx, digestive system, respiratory system, bones and joints, soft tissue including heart, skin excluding basal and squamous, breast, female genital system, male genital system, urinary system, eye and orbit, brain and other nervous system, endocrine system, lymphoma, myeloma, leukemia, mesothelioma and Kaposi sarcoma. Other undefined primary tumors were classified into 'Miscellaneous' group. The classification of primary cancer was summarized in Supplementary Table 1.

 Based on the International Statistical Classification of Diseases and Related Health Problems, Tenth Revision (ICD-10) codes, the non-cancer COD was classified into thirteen categories, which was listed in Supplementary Table 2.

### Statistics analysis

Mortality rate based on cancer incidence, namely incidence-based mortality (IBM), was calculated to avoid the effect of cancer onset. Furthermore, to reduce the confounding effect of age, the mortality rate was adjusted by age based on the corresponding age groups of the 2000 US standard population. Displayed by calendar year, annual percent change (APC) of mortality was calculated and used to measure change in rates over time. Based on Monte Carlo permutation method, trend data were analyzed and apparent change in trend with statistically significance can be outputted in tables and visualized in graphs^8^. The analyses were performed in Joinpoint Trend Analysis Software (Version 4.9.1.0).

Standardized mortality ratio (SMR) was calculated as the ratio of observed-to-expected deaths. The expected deaths represented patients in a demographically similar population who was expected to die, and the data can be calculated using SEER software directly. Surveillance Research Program, National Cancer Institute SEER*Stat software (version 8.4.0.) was used to extracted raw data while GraphPad Prism software (version 8.0.2) and R software (version 4.1.2) were conducted for data visualization.

## Results

### The frequencies of deaths among different age groups and percentage of COD

**Figure 1:**
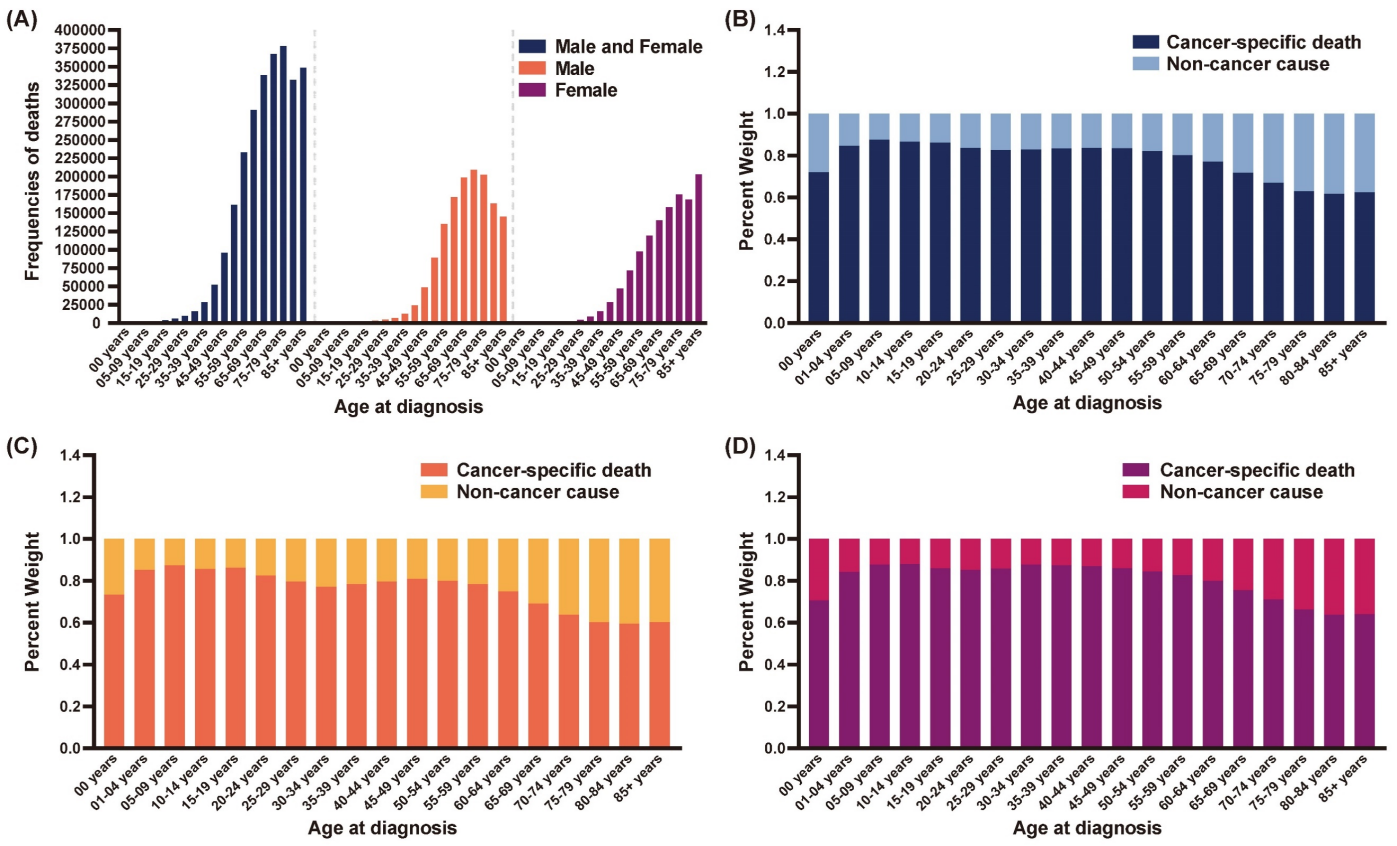
The frequencies of deaths and percent weight of cause of death (COD) among different age groups. (A) The frequencies of deaths in the total cohort, male patients and female patients; (B) The percent weight of cancer-specific death and non-cancer cause in the total cohort; (C) The percent weight of cancer-specific death and non-cancer cause in male patients; (D) The percent weight of cancer-specific death and non-cancer cause in female patients.

Initially, a total of 5,580,217 malignant cancer patients were extracted from SEER database with 2,909,814 cases alive at the last follow-up (Table 1). As shown in Figure 1A and Table 1, there were 2,670,403 deaths recorded between 2010 and 2019 totally, with 1,424,057 for male and 1,246,346 for female patients respectively. A total of 1,876,179 patients were died of cancer-specific cause while other 794,224 cases were recorded as non-cancer caused death. The frequencies of patients among different age groups were summarized in Table 1. Patients with age older than 65 years accounted for more than one half (53.6%). Among them, 75-79 years group contributed the most deaths (378,231 death cases), followed by 70-74 years group (367,011 death cases), 85+ years group (348,601 death cases) and 65-69 years group (338,239 death cases). As for data stratified by gender, 75-79 years group and 85+ years group possessed the most deaths for male (209,117 death cases) and female (202,969 death cases), respectively. Generally, the number of deaths ascended with older age of cancer diagnosis.

For patients older than 50 years, the proportion of cases died of non-cancer causes increased with growing age (Figure 1B). As for patients older than 70 years, more than thirty percent died due to non-cancer causes. The proportions for non-cancer COD, in descending order, were: 38.3%, 37.6%, 37.0% and 33.1% for 80-84 years group, 85+ years group, 75-79 years group and 70-74 years group, respectively. As shown in Figure 1C and Figure 1D, the same trends were illustrated in both male and female patients. Noteworthily in 0 years group, the non-cancer COD accounted for more than one quarter of total deaths, which might be attributed to severe complications in neonatal period and infancy.

### The primary cancers among different age groups

As presented in Supplementary Figure 1, digestive system cancer (688,953 death cases), respiratory system cancer (607,521 death cases), male genital system cancer (236,258 death cases), breast cancer (211,849 death cases) and urinary system cancer (164,355 death cases) were the five most common primary cancer types totally. Meanwhile, there were 2,924, 3,224 and 4,821 recorded deaths in Kaposi sarcoma, eye and orbit cancer, bones and joints cancer respectively, which were the three fewest types for total death. In deceased patients younger than 19 years at diagnosis, leukemia and cancer on brain and other nervous system were two common tumor types. The sum of these two types accounted for more than one half of total death in 0 years, 1-4 years, 5-9 years and 10-14 years group. The percentage was up to nearly 40.0% in 15-19 years group. For patients with age older than 25 years, digestive system cancer was the most common primary cancer until its dominated role was surpassed by respiratory system cancer for patients older than 65 years old.

The proportions of primary tumors were heterogeneous across gender and age groups, which presented in the diagram (Supplementary Figure 2 for male and Supplementary Figure 3 for female, respectively). As for male gender, digestive system cancer (382,657 death cases), respiratory system cancer (337,550 death cases) and male genital system cancer (236,258 death cases) were top three cancer types. While in female gender, digestive system cancer (306,296 death cases), respiratory system cancer (269,971 death cases) were the most common cancer types, followed by breast cancer (209,642 death cases) ranking third.

### Age-adjusted mortality rate among different age groups and trend analyses

The modeled age-adjusted mortality rate per 100,000 persons among different age groups was recorded in Supplementary Table 3. As shown in Table 2, the mortality rate increased gradually in total cohort, by 5.6% annually (95%CI: 4.9 to 6.4) in total death, 4.0% annually (95%CI: 2.8 to 5.3) in cancer-specific death and 10.9% annually (95%CI: 9.9 to 12.1) in non-cancer COD over the study period.

**Figure 2:**
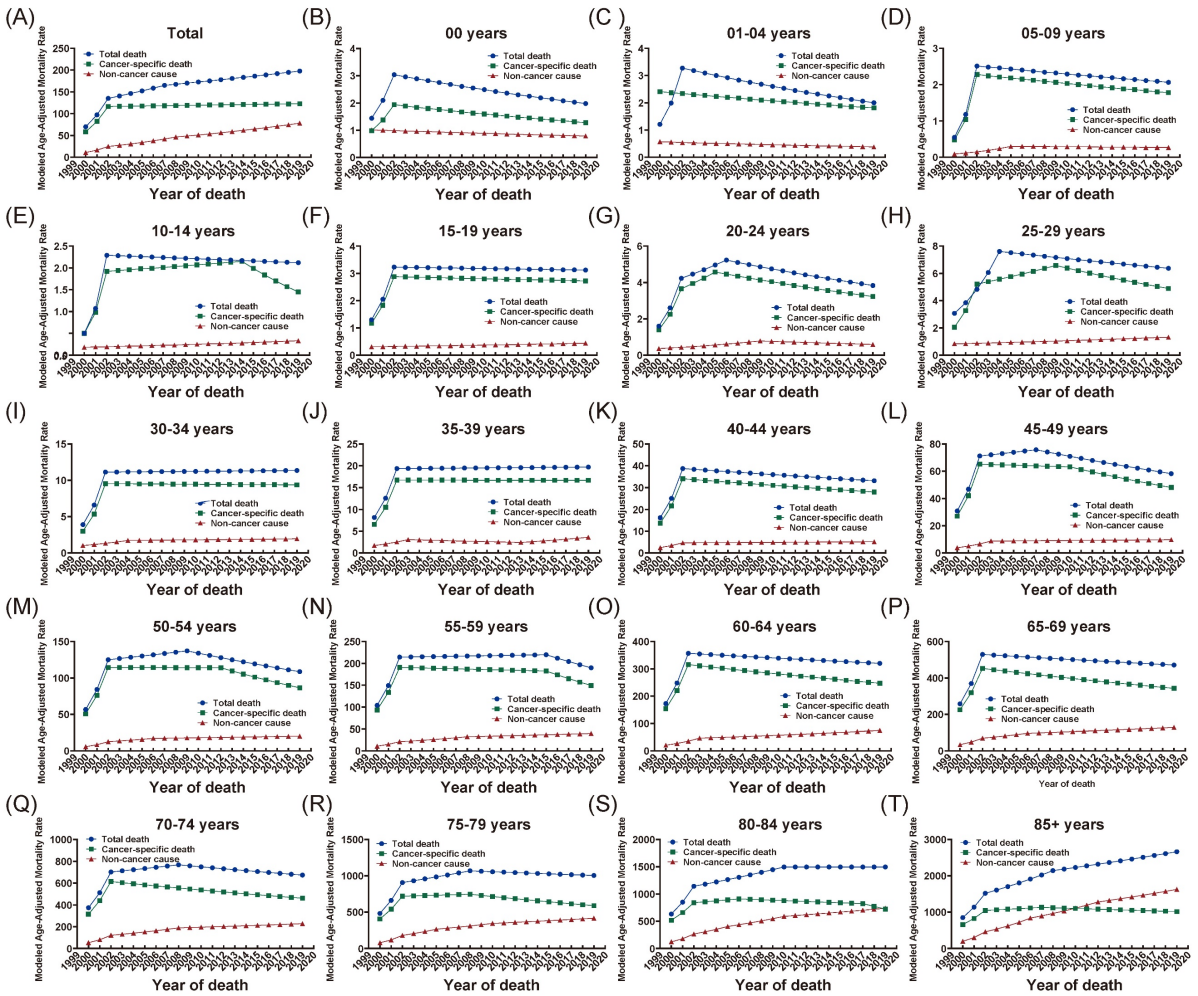
Trends in age-adjusted mortality rate from 2000 to 2019 among different age groups by total death, cancer-specific death and non-cancer caused death. X axis represented year of death while Y axis represented modeled age-adjusted mortality rate per 100,000 persons.

The trend of mortality rates per 100,000 persons were presented in Figure 2 and the joint point occurred normally in three periods: 2002-2003, 2006-2008 and 2010. As for patients in 30-34 years and 35-39 years group, both total mortality and cancer specific mortality increased substantially from 2000 to 2002 and kept steady subsequently. In 40 to 49 years groups, the same pronounced increases were seen in total death and cancer-specific death while the rates gradually decreased after 2002. For patients older than 50 years, the average annual percent change (AAPC) of non-cancer caused mortalities were up to larger than 5.0% and increased with age. The AAPC for 50-54 years group, 55-59 years group, 60-64 years group, 65-69 years group, 70-74 years group, 75-79 years group, 80-84 years group and 85+ years group were 6.8% (95%CI: 3.3 to 10.5), 7.1% (95%CI: 3.9 to 10.3), 7.0% (95%CI: 4.9 to 9.1), 7.2% (95%CI: 5.2 to 9.3), 7.9% (95%CI: 6.5 to 9.3), 9.2% (95%CI: 7.9 to 10.5), 9.9% (95%CI: 8.7 to 11.2) and 11.7% (95%CI: 10.3 to 13.1), respectively (Table 2). The annual percent change (APC) of age-adjusted mortality rates for total death, cancer-specific death and non-cancer caused death were presented in Supplementary Table 4, Supplementary Table 5 and Supplementary Table 6 respectively.

### Age distribution of deaths in different primary cancers

**Figure 3:**
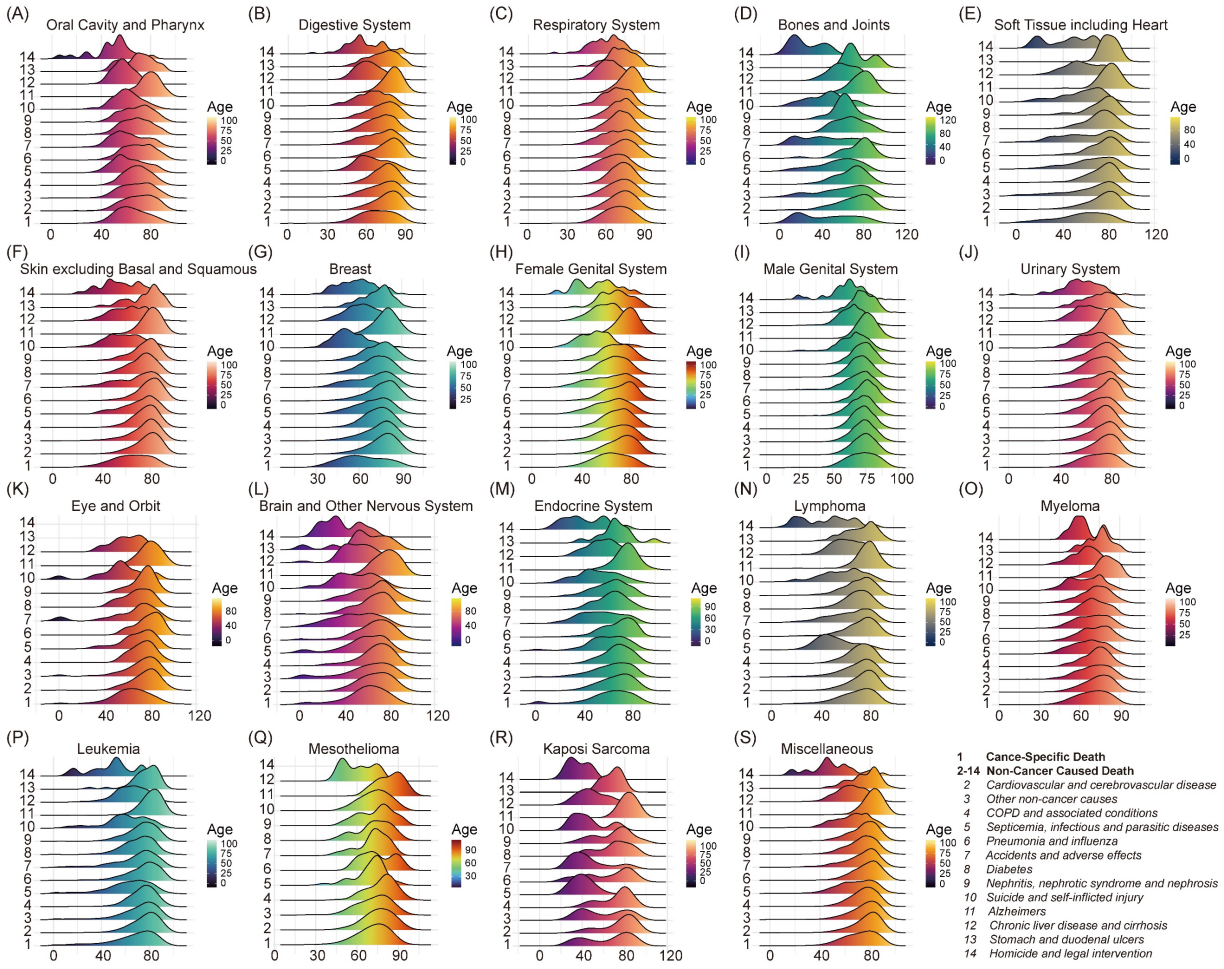
Age distribution of different cause of death (COD) among primary tumors. X axis represented year of diagnosis and Y axis represented different COD. The unique number in Y axis indicated different types of COD while the illustration was presented on lower right corner of the figure.

For each type of primary tumors, the age distribution of cancer-specific death and thirteen non-cancer caused death were visualized in ridgeline plot (Figure 3). The code 1-14 at the bottom right of the Figure 3 represented the several different causes of death correspondingly (code 1 for cancer-specific death and code 2-14 for thirteen non-cancer caused death). As for cancer-specific death, different mean age and age distribution were seen among primary tumor types, which caused by prone age and prognosis of cancer.

 In majority of tumors, the skewed distribution of age was observed in patients dead attributed to the chronic non-infectious disease such as cardiovascular and cerebrovascular disease, COPD and associated conditions, pneumonia and influenza, diabetes, chronic liver disease and cirrhosis as well as Alzheimer. More deaths tended to occur in older patients. On the other hand, Kaposi sarcoma was the exception. In Kaposi sarcoma, distribution of younger age was presented in septicemia, infectious and parasitic diseases, accidents and adverse effects, suicide and self-inflicted injury, and homicide and legal intervention. The mean age of four types non-cancer caused death were 40.70 (95%CI: 40.18-41.21), 46.57 (95%CI: 41.07-52.08), 45.78 (95%CI: 37.77-53.79) and 36.20 (95%CI: 24.05-48.35), respectively.

### Observed deaths and standardized mortality ratios (SMRs)

The total cohort (all site tumors) and seven major types of primary tumors (digestive system cancer, respiratory system cancer, breast cancer, male genital system cancer, female genital system cancer, urinary system cancer, brain and other nervous system cancer) were further studied. The five non-cancer COD types with the most observed deaths were focused and standardized mortality ratios (SMRs) were calculated accordingly.

**Figure 4:**
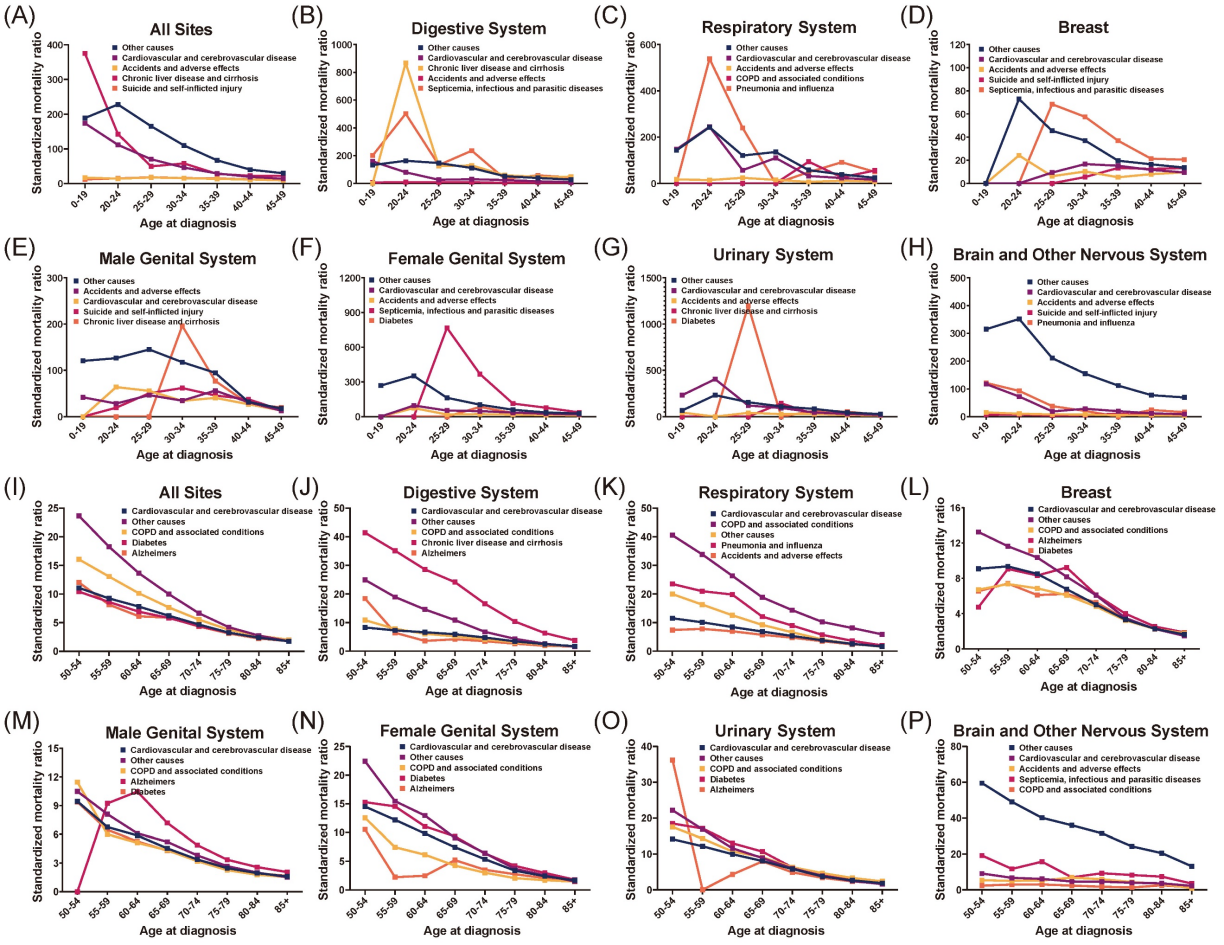
The standardized mortality ratios (SMRs) in all site tumors and seven major types of tumors (for patients younger than fifty years, and old older than fifty years old). The top five non-cancer cause of death (COD) in each tumor type was analyzed and visualized.

As for patients younger than 50, the standardized mortality ratios (SMRs) of top five non-cancer COD in both total cohort and each primary tumor were plotted in Figure 4 (Figure 4A for all tumor sites and Figure 4B-H for different primary tumor types). Cardiovascular and cerebrovascular disease was identified as the predominant non-cancer COD, ranking second after undefined other cause in almost every tumor type except for male genital system cancer. The other two common non-cancer causes were accidents and adverse effects, and suicide and self-inflicted injury, and the SMRs of these two were stable across different age groups. Besides, disease-specific COD was showed, such as chronic liver disease and cirrhosis in digestive system, and pneumonia and influenza in respiratory system cancer.

Chronic non-infectious disease and Alzheimer were the main non-cancer COD in older population. As shown in Figure 4I, the top five non-cancer COD were cardiovascular and cerebrovascular disease (SMR: 2.58; 95%CI: 2.57-2.58), other causes (SMR: 3.12; 95%CI: 3.11-3.13), COPD and associated conditions (SMR: 3.29; 95%CI: 3.26-3.31), diabetes (SMR: 3.00; 95%CI: 2.96-3.04) as well as Alzheimer (SMR: 2.02; 95%CI: 2.00-2.04). The SMRs of these causes were higher among younger patients and gradually dropped in older age groups. In all site tumors, the SMR of COPD and associated conditions was up to 16.05 (95%CI: 14.86-17.31) in 50-54 years group. While the SMRs in 55-59 years group, 60-64 years group, 65-69 years group, 70-74 years group, 75-79 years group, 80-84 years group and 85+ years group were 13.06 (95%CI: 12.44-13.70), 10.13 (95%CI: 9.80-10.47), 7.66 (95%CI: 7.48-7.85), 5.54 (95%CI: 5.44-5.65), 3.76 (95%CI: 3.70-3.83), 2.73 (95%CI: 2.68-2.77) and 2.01 (95%CI: 1.98-2.04), respectively. The decrease of SMR was conventional among tumor types in patients older than 50 years (Figure 4I-F).

## Discussion

Malignant cancer was one of the most common causes of death globally. In the United States, heart disease was the leading cause of death, followed by cancer, accidents (unintentional injuries), chronic lower respiratory diseases, cerebrovascular disease, and Alzheimer's disease^1^. According to the global cancer statistics in 2020, the global burden of cancer was expected to be 28.4 million cases in 2040, around 47% raise since 2020. Over the past decades, the profile of the decreasing and increasing cancer types changed gradually. The previous literature revealed that the cancer profile in China and the United States were converging. The burden of liver, stomach, and esophagus decreased, while that of the lung, colorectum, breast, and prostate increased. The human development index (HDI) had an impact on cancer incidence and projected global cancer burden. Transitioning (low and medium HDI) countries/regions might have a larger increase when compared with transitioned (high and very high HDI) due to the demographic changes^9^, ^10^. Due to the development of diagnosis and treatment techniques, the survival of patients diagnosed with the most cancer types was improved. Change in patients' life-span after diagnosis for non-cancer-related comorbidities promoted a positive association with their overall survival. By analyzing the COD of cancer patients, patients who were at high risk of dying for some specific reasons may be detected early and may benefit from the targeted preventive measures.

Population aging played a growing determinant role in the incremental cancer burden^11^. Based on cancer statistics in 2022, data showed that patients aged 60-79 contributed the most deaths, followed by the elder patients (≥80). This trend was consistent in both male and female patients. Contributed deaths ascended with increased age^1^. Among the participants included in the current study, the similar findings were observed in both genders. With the growing age, the proportion of cases that died of non-cancer causes increased. Modeled age-adjusted mortality rate per 100,000 persons revealed that the mortality rate increased by 5.6% annually in total death, 4.0% annually in cancer-specific death, and 10.9% annually in non-cancer COD over the study period. This indicated a predominant role of non-cancer COD contributed to the increasement in the total death. In addition, the current study reported an increasing trend of mortality among patients older than 50 years, since the AAPC of non-cancer-caused mortalities was up to larger than 5.0% and increased with age. In patients with colorectal cancer who underwent curative surgery, age was an independent prognostic factor which significantly restricted the overall survival^12^. Cardiovascular disease was a noticeable non-cancer COD among patients diagnosed with colorectal cancer^13^-^15^. The mortality risk of colorectal patients died of non-cancer causes was raised with the increasing age and longer follow-up time^15^. This phenomenon can be explained as the older patients were easily to be with comorbidities, including heart disease, chronic lower respiratory diseases, cerebrovascular disease, and Alzheimer's disease. Meanwhile, physicians might have a relatively conservative attitude toward treating elderly patients diagnosed with cancer. In particular, decisions on surgery time and chemotherapy dose were made prudently, because the elderly patients may be prone to die of comorbidities but not cancer.

Distant metastasis formation was a major COD in most patients with cancer. Of the total deaths among patients with metastatic prostate cancer, 16.7% of patients died of non-cancer causes. The most common non-cancer COD were cardiovascular diseases, followed by COPD, and cerebrovascular diseases^13^. Beyond the malignant nature of cancer, other concurrent disease or condition also contributed to the decease of cancer patients. Our previous analysis showed that non-cancer causes covered 6.9% of patients diagnosed with malignant cancer with synchronous bone metastases. Common non-cancer COD included cardiovascular and cerebrovascular diseases, COPD and associated conditions, and septicemia, infectious and parasitic diseases^5^. The population-based cohort study based on the SEER database focusing on the COD during prostate cancer survivorship reported, the risk for death from most other non-cancer causes of men with distant prostate cancer was heightened. Particularly, the SMR for cardiac-related death was 1.48 (95% CI, 1.41-1.54) and for suicide was 2.32 (95% CI, 1.78-2.96)^16^.

A systematic review and meta-analysis indicated the suicide mortality rate (pooled SMR = 1.55; 95% CI = 1.37-1.74) in cancer patients. The SMR in male (pooled SMR = 1.67; 95% CI = 1.48-1.89) and female patients (SMR = 1.34; 95% CI = 1.20-1.50) showed the elevated relative suicide mortality risk^17^. Association between diagnosis of male genital system cancer, including prostate cancer and testis cancer, and death from suicide was observed previously^6^, ^18^-^20^. The SMR of suicide increased gradually with age and peaked among patients in the age 30-34 group in our study. Men in this period were particularly notable unmarried. Our finding was conventional with previous work. The underlying reason for this may be related to the subsequent negative mental health impact after diagnosis of genital cancer. Depressive, anxiety and suicidal ideation were common among patients with prostate cancer^21^. Increased incidence of mental health change might be due to the androgen deprivation therapy^22^, ^23^. This risk of depression and inpatient psychiatric treatment increased with a longer duration of androgen deprivation therapy^23^.

In the total cohort enrolled in the current study, digestive system cancer and respiratory system cancer were the two most common primary cancer types. As for the male gender, the third caner type was male genital system cancer. Breast cancer ranked the third for female gender. In China, the top 3 cancer types in 2018 by incidence were lung, stomach, and colorectum cancer. While in Chinese females, the top 3 cancer types by incidence were breast, lung, and colorectum stomach cancer^24^. An epidemiology analysis suggested that the estimated leading five cancers diagnosed in 2022 would be cancers in the lung, colorectum, stomach, liver, and breast in China, and cancers in the breast, lung, prostate, colorectum, and melanoma of skin in the USA. The leading five causes of cancer death between China and USA showed different, the cancers in the lung, liver, stomach, esophagus, and colorectum in China, and cancers of the lung, colorectum, pancreas, breast, and prostate in the USA^11^. Although there were disparities in the disease burden globally, it can be concluded that the cancer of digestive and respiratory system had a major proportion leading to the burden. For patients enrolled in this study, the digestive system cancer was the most common primary cancer death among patients with age older than 25 years. As to patients older than 65 years old, the dominate role of the digestive cancer was surpassed by the respiratory system cancer. Lung cancer took decades to develop after the initiation of smoking and thus peaks in the elderly^25^. Although new cases and cancer-associated deaths occurred in young adults (20-39 years old) were lower than 2.60%, the digestive cancer accounted for 12.24% in all new cancer cases and 25.26% of all cancer-associated deaths in this group population^26^. What could be concluded from the above data was a heavy digestive system cancer burden and poor outcome. Primary and secondary prevention strategies for the above two cancer types need to be reinforced. The environmental exposure (air pollution^27^, household air pollution from solid fuels^28^) and lifestyle (unrestrained eating behavior^29^ and smoking^30^, etc) factors were supposed to be the main risk factors attributed to heavy burden of lung cancer and digestive system cancer. Adopting healthy lifestyles, which contributed the substantial lower risk in cancer morbidity and mortality, should be encouraged^31^.

Previous studies reported multiple causes of death (COD) in single type of cancer (e.g. prostate^32^ and breast^33^), or reported the particular cause of death among several cancer types (e.g. suicide^34^). In current study, we conducted a comprehensive analysis among all cancer patients died of various causes from 2010 to 2019. After the year of 2010, a series of treatments were developed rapidly, such as targeted therapy and immunotherapy^35^. Thus, the prognosis of cancer patients was significantly improved. Subsequently, compared with trend before 2010, the mortality and COD in cancer patients were different. Based on the latest updated 2010-2019 data from SEER database, the trend can be explored. Our present study characterized the distribution of 14 COD among 18 individual primary tumors, as a function of patient age. According evidenced by our study and a comparison with the most recent published "Cancer statistics, 2022"^1^, the primary causes of cancer in the United States included cardiovascular and cerebrovascular disease, accidents and adverse effects, suicide and self-inflicted injuries. Furthermore, our research revealed that cancer patients largely died of heart disease and “other causes”. Compared with the young patients, the elder patients mostly died by comorbidities such as diabetes, cardiovascular and cerebrovascular diseases. It was acknowledged that physicians could not ignore that cardiovascular and cerebrovascular diseases could also be an important non-cancer COD even in young cancer patients.

This study has some limitations. There was a possibility of wrong/inappropriate classification of COD which was inherent to epidemiologic research. Globally, China had the largest number of cancer deaths, followed by India and the United States^36^. Data included in the current study was from the SEER database. To some content, the findings only presented the fact and future trend of United States, but might not able to be adopted to every country worldwide. Geographic characteristics investigation needs the support of local large-scale epidemiology data. The current study was a large-scale study with detailed COD records and comprehensive analysis of death among patients with one primary cancer.

## Conclusion

Cancer burden remained high worldwide. The substantial mortality and COD varied across age and primary cancer types. Both cancer-specific death and non-cancer COD were systemically analyzed in this study and comorbidities were unignored COD among cancer patients. Non-cancer COD was the important indicator when establishing cancer prevention and management systems. The current study can provide the guidance for the physicians on treatment decision-making in cancer patients at different age.

## Supplementary Material

Supplementary figures and tables 1, 2, 4, 5, 6.

Supplementary table 3.

## Figures and Tables

**Table 1:** The frequencies of recorded cases among different age groups.

Age group	Alive	Total death	Cancer-specific death	Non-cancer caused death
00 years	4,105	1,089	784	305
01-04 years	15,986	2,715	2,300	415
05-09 years	11,227	2,192	1,920	272
10-14 years	12,581	2,714	2,350	364
15-19 years	20,360	4,094	3,524	570
20-24 years	33,044	5,981	4,999	982
25-29 years	51,678	9,476	7,823	1,653
30-34 years	76,390	15,932	13,219	2,713
35-39 years	107,532	28,275	23,569	4,706
40-44 years	160,095	52,479	43,868	8,611
45-49 years	232,841	95,920	80,005	15,915
50-54 years	326,818	161,282	132,267	29,015
55-59 years	400,857	233,130	186,910	46,220
60-64 years	430,693	290,873	224,064	66,809
65-69 years	412,996	338,239	242,586	95,653
70-74 years	293,318	367,011	245,488	121,523
75-79 years	178,295	378,231	238,141	140,090
80-84 years	90,144	332,169	204,834	127,335
85+ years	50,854	348,601	217,528	131,073
	2,909,814	2,670,403	1,876,179	794,224

**Table 2:** Average annual percent change (AAPC) of age-adjusted mortality rate among different age groups.

Age	Classification	AAPC	Lower CI	Upper CI	Test Statistic	*P-Value*
**Total**	Total death	5.6*	4.9	6.4	14.8	*< 0.001*
	Cancer-specific death	4.0*	2.8	5.3	6.5	*< 0.001*
	Non-Cancer cause	10.9*	9.9	12.1	20.5	*< 0.001*
**00 years**	Total death	1.7	-2.4	5.9	0.8	*0.422*
	Cancer-specific death	1.4	-3.3	6.4	0.6	*0.558*
	Non-Cancer cause	-1.3	-4.2	1.6	-1.0	*0.346*
**01-04 years**	Total death	2.7	-2.7	8.4	1.0	*0.340*
	Cancer-specific death	-1.5	-3.4	0.5	-1.6	*0.127*
	Non-Cancer cause	-2.1	-4.9	0.8	-1.5	*0.149*
**05-09 years**	Total death	7.2*	1.7	12.9	2.6	*0.009*
	Cancer-specific death	7.1*	1.4	13.2	2.5	*0.013*
	Non-Cancer cause	5.9	-0.4	12.6	1.8	*0.068*
**10-14 years**	Total death	7.9*	0.7	15.5	2.2	*0.031*
	Cancer-specific death	5.8	-0.4	12.3	1.8	*0.070*
	Non-Cancer cause	3.2*	0.1	6.3	2.2	*0.045*
**15-19 years**	Total death	4.7*	1.6	8.0	2.9	*0.003*
	Cancer-specific death	4.6*	1.2	8.0	2.7	*0.007*
	Non-Cancer cause	1.8	-0.5	4.2	1.7	*0.115*
**20-24 years**	Total death	4.7*	2.4	7.1	4.0	*< 0.001*
	Cancer-specific death	4.5*	1.1	8.1	2.6	*0.009*
	Non-Cancer cause	2.6	-0.4	5.7	1.7	*0.088*
**25-29 years**	Total death	3.9*	1.3	6.6	2.9	*0.003*
	Cancer-specific death	4.7*	1.9	7.6	3.3	*0.001*
	Non-Cancer cause	2.5*	0.8	4.2	3.1	*0.007*
**30-34 years**	Total death	5.8*	3.4	8.2	4.9	*< 0.001*
	Cancer-specific death	6.2*	3.7	8.7	5.0	*< 0.001*
	Non-Cancer cause	3.4*	0.7	6.2	2.5	*0.013*
**35-39 years**	Total death	4.7*	3.4	6.1	7.1	*< 0.001*
	Cancer-specific death	5.0*	3.0	7.1	5.0	*< 0.001*
	Non-Cancer cause	4.0*	1.0	7.0	2.7	*0.008*
**40-44 years**	Total death	3.8*	2.5	5.2	5.8	*< 0.001*
	Cancer-specific death	3.8*	2.3	5.4	4.9	*< 0.001*
	Non-Cancer cause	3.9*	1.2	6.6	2.9	*0.004*
**45-49 years**	Total death	3.4*	2.4	4.4	6.7	*< 0.001*
	Cancer-specific death	3.1*	2.0	4.1	5.7	*< 0.001*
	Non-Cancer cause	5.0*	2.3	7.7	3.7	*< 0.001*
**50-54 years**	Total death	3.5*	2.3	4.6	6.1	*< 0.001*
	Cancer-specific death	2.9*	1.8	4.0	5.2	*< 0.001*
	Non-Cancer cause	6.8*	3.3	10.5	3.9	*< 0.001*
**55-59 years**	Total death	3.2*	2.3	4.2	6.7	*< 0.001*
	Cancer-specific death	2.5*	1.4	3.6	4.5	*< 0.001*
	Non-Cancer cause	7.1*	3.9	10.3	4.5	*< 0.001*
**60-64 years**	Total death	3.3*	2.5	4.1	8.3	*< 0.001*
	Cancer-specific death	2.5*	1.4	3.6	4.6	*< 0.001*
	Non-Cancer cause	7.0*	4.9	9.1	6.8	*< 0.001*
**65-69 years**	Total death	3.2*	2.0	4.4	5.3	*< 0.001*
	Cancer-specific death	2.2*	0.8	3.7	3.1	*0.002*
	Non-Cancer cause	7.2*	5.2	9.3	7.1	*< 0.001*
**70-74 years**	Total death	3.2*	2.2	4.1	6.9	*< 0.001*
	Cancer-specific death	2.0*	0.7	3.4	2.9	*0.003*
	Non-Cancer cause	7.9*	6.5	9.3	11.4	*< 0.001*
**75-79 years**	Total death	3.9*	3.1	4.8	9.1	*< 0.001*
	Cancer-specific death	2.0*	0.9	3.1	3.4	*0.001*
	Non-Cancer cause	9.2*	7.9	10.5	14.3	*< 0.001*
**80-84 years**	Total death	4.6*	3.7	5.6	10.0	*< 0.001*
	Cancer-specific death	1.8*	1.3	2.3	7.1	*< 0.001*
	Non-Cancer cause	9.9*	8.7	11.2	16.7	*< 0.001*
**85+ years**	Total death	6.2*	5.1	7.3	11.2	*< 0.001*
	Cancer-specific death	2.3*	0.9	3.8	3.1	*0.002*
	Non-Cancer cause	11.7*	10.3	13.1	17.3	*< 0.001*

* Indicates that the AAPC is significantly different from zero at the alpha = 0.05 level.
