# Supplementary material for: Age-related Disparities in Pan-Cancer Mortality and Causes of Death: Analysis of Surveillance, Epidemiology, and End Results (SEER) Data
Source: J Cancer. 2024 Jan 21;15(6):1613–23. doi: 10.7150/jca.91758 (PMC10869975; doi:10.7150/jca.91758)

Supplementary Table 1. The classification of primary cancer in the present study.

Site Group in SEER database	Classification in the current study	Including
**Oral Cavity and Pharynx**	Oral Cavity and Pharynx	Lip, Tongue, Salivary Gland, Floor of Mouth, Gum and Other Mouth, Nasopharynx, Tonsil, Oropharynx, Hypopharynx, Other Oral Cavity and Pharynx
**Digestive System**	Esophagus	
	Stomach	
	Small Intestine	
	Colon and Rectum	Cecum, Appendix, Ascending Colon, Hepatic Flexure, Transverse Colon, Splenic Flexure, Descending Colon, Sigmoid Colon, Large Intestine, NOS, Rectosigmoid Junction, Rectum
	Anus, Anal Canal and Anorectum	
	Liver	
	Intrahepatic Bile Duct	
	Gallbladder	
	Other Biliary	
	Pancreas	
	Retroperitoneum	
	Peritoneum, Omentum and Mesentery	
	Other Digestive Organs	
**Respiratory System**	Lung and Bronchus	
	Respiratory System except for Lung and Bronchus	Nose, Nasal Cavity and Middle Ear; Larynx; Pleura; Trachea, Mediastinum and Other Respiratory Organs
**Bones and Joints**	Bones and Joints	
**Soft Tissue including Heart**	Soft Tissue including Heart	
**Skin excluding Basal and Squamous**	Skin excluding Basal and Squamous	

**Breast**	Breast	
**Female Genital System**	Cervix Uteri	
	Corpus and Uterus, NOS	Corpus Uteri; Uterus, NOS
	Ovary	
	Other Female Genital Organs	Vagina; Vulva; Other Female Genital Organs
**Male Genital System**	Prostate	
	Other Male Genital Organs	Testis; Penis; Other Male Genital Organs
**Urinary System**	Urinary Bladder	
	Kidney and Renal Pelvis	
	Ureter	
	Other Urinary Organs	
**Eye and Orbit**	Eye and Orbit	
**Brain and Other Nervous System**	Brain and Other Nervous System	
**Endocrine System**	Thyroid	
	Other Endocrine including Thymus	
**Lymphoma**	Hodgkin Lymphoma	
	Non-Hodgkin Lymphoma	
**Myeloma**	Myeloma	
**Leukemia**	Leukemia	
**Mesothelioma**	Mesothelioma	
**Kaposi Sarcoma**	Kaposi Sarcoma	
**Miscellaneous**	Miscellaneous	

Supplementary Table 2. The classification of non-cancer cause of death and associated ICD-10 codes.

Classification	Cause of death definition	ICD-10 corresponding codes
**Cardiovascular and cerebrovascular disease**	Diseases of Heart	I00-I09, I11, I13, I20-I51
	Hypertension without Heart Disease	I10, I12
	Cerebrovascular Diseases	I60-I69
	Atherosclerosis	I70
	Aortic Aneurysm and Dissection	I71
	Other Diseases of Arteries, Arterioles, Capillaries	I72-I78
**Other causes**	In situ, benign or unknown behavior neoplasm	D00-D48
	Complications of Pregnancy, Childbirth, Puerperium	A34, O00-O95, O98-O99
	Congenital Anomalies	Q00-Q99
	Certain Conditions Originating in Perinatal Period	P00-P96
	Symptoms, Signs and Ill-Defined Conditions	R00-R99
	Other Cause of Death	-
**COPD and associated conditions**	Chronic Obstructive Pulmonary Disease and Allied Cond	J40-J47
**Septicemia, infectious and parasitic diseases**	Tuberculosis	A15-A19
	Syphilis	A50-A53
	Septicemia	A40-A41
	Other Infectious and Parasitic Diseases including HIV	A00-A08, A20-A33, A35-A39, A42-A49, A54-B19, B25-B99
**Pneumonia and influenza**	Pneumonia and influenza	J09-J18
**Accidents and adverse effects**	Accidents and adverse effects	V01-X59, Y85-Y86

**Diabetes**	Diabetes Mellitus	E10-E14
**Nephritis, nephrotic syndrome and nephrosis**	Nephritis, nephrotic syndrome and nephrosis	N00-N07, N17-N19, N25-N27
**Suicide and self-inflicted injury**	Suicide and self-inflicted injury	U03, X60-X84, Y87.0
**Alzheimers**	Alzheimers (ICD-9 and 10 only)	G30
**Chronic liver disease and cirrhosis**	Chronic liver disease and cirrhosis	K70, K73-K74
**Stomach and duodenal ulcers**	Stomach and duodenal ulcers	K25-K28
**Homicide and legal intervention**	Homicide and legal intervention	U01-U02, X85-Y09, Y35, Y87.1, Y89.0

Supplementary Table 4. Annual percent change (APC) of age-adjusted mortality rates for total death.

Age	Segment	Lower Endpoint	Upper Endpoint	APC	Lower CI	Upper CI	Test Statistic (t)	*Prob >/t/*
**Total**	1	2000	2002	38.9*	30.1	48.2	11	*< 0.001*
	2	2002	2007	4.1*	2.5	5.6	5.9	*< 0.001*
	3	2007	2019	1.5*	1.3	1.8	13.5	*< 0.001*
**00 years**	1	2000	2002	45	-3.4	117.8	1.9	*0.070*
	2	2002	2019	-2.5*	-3.7	-1.3	-4.3	*0.001*
**01-04 years**	1	2000	2002	64.3	-5	184.2	1.9	*0.073*
	2	2002	2019	-2.8*	-4.2	-1.5	-4.4	*< 0.001*
**05-09 years**	1	2000	2002	113.2*	25.3	262.8	3	*0.008*
	2	2002	2019	-1.1*	-2.2	-0.1	-2.4	*0.033*
**10-14 years**	1	2000	2002	113.7*	6	330.9	2.3	*0.036*
	2	2002	2019	-0.5	-1.8	0.8	-0.8	*0.460*
**15-19 years**	1	2000	2002	57.8*	15.5	115.7	3.1	*0.007*
	2	2002	2019	-0.2	-0.9	0.5	-0.6	*0.583*
**20-24 years**	1	2000	2002	62.8*	33.3	99	5.3	*< 0.001*
	2	2002	2006	5.4	-0.9	12.1	1.9	*0.085*
	3	2006	2019	-2.4*	-3	-1.7	-8	*< 0.001*
**25-29 years**	1	2000	2004	25.5*	10.6	42.3	3.8	*0.002*
	2	2004	2019	-1.2*	-2.3	0	-2.1	*0.049*
**30-34 years**	1	2000	2002	68.7*	34.3	111.8	4.9	*< 0.001*
	2	2002	2019	0.1	-0.4	0.6	0.5	*0.643*
**35-39 years**	1	2000	2002	54.0*	35.4	75.2	7.1	*< 0.001*
	2	2002	2019	0.1	-0.2	0.4	0.7	*0.505*
**40-44 years**	1	2000	2002	54.6*	36	75.7	7.3	*< 0.001*

	2	2002	2019	-0.9*	-1.2	-0.6	-5.9	*< 0.001*
**45-49 years**	1	2000	2002	51.8*	38.9	66	10.2	*< 0.001*
	2	2002	2007	1.2	-0.6	3.2	1.4	*0.177*
	3	2007	2019	-2.2*	-2.5	-1.8	-12.7	*< 0.001*
**50-54 years**	1	2000	2002	48.5*	33.6	65.1	8.2	*< 0.001*
	2	2002	2009	1.3*	0.1	2.5	2.4	*0.032*
	3	2009	2019	-2.3*	-2.8	-1.8	-9.2	*< 0.001*
**55-59 years**	1	2000	2002	43.6*	31.3	57.1	8.8	*< 0.001*
	2	2002	2015	0.2	-0.1	0.5	1.3	*0.234*
	3	2015	2019	-3.6*	-5.2	-1.9	-4.6	*0.001*
**60-64 years**	1	2000	2002	43.7*	33	55.4	9.9	*< 0.001*
	2	2002	2019	-0.6*	-0.8	-0.5	-7.9	*< 0.001*
**65-69 years**	1	2000	2002	43.3*	27.4	61.3	6.5	*< 0.001*
	2	2002	2019	-0.7*	-1	-0.4	-5.4	*< 0.001*
**70-74 years**	1	2000	2002	37.1*	26.4	48.7	8.5	*< 0.001*
	2	2002	2008	1.5*	0.1	2.9	2.3	*0.038*
	3	2008	2019	-1.2*	-1.5	-0.8	-7	*< 0.001*
**75-79 years**	1	2000	2002	37.1*	27	48	9	*< 0.001*
	2	2002	2008	2.8*	1.4	4.1	4.6	*0.001*
	3	2008	2019	-0.6*	-0.9	-0.2	-3.4	*0.005*
**80-84 years**	1	2000	2002	34.3*	23.4	46.3	7.5	*< 0.001*
	2	2002	2010	3.4*	2.6	4.3	9.2	*< 0.001*
	3	2010	2019	0	-0.5	0.5	0	*1*
**85+ years**	1	2000	2002	33.6*	20.9	47.6	6.3	*< 0.001*
	2	2002	2008	5.9*	4.3	7.5	8.2	*< 0.001*
	3	2008	2019	2.0*	1.6	2.3	12.5	*< 0.001*

* Indicates that the Annual Percent Change (APC) is significantly different from zero at the alpha = 0.05 level.

Supplementary Table 5. Annual percent change (APC) of age-adjusted mortality rates for cancer-specific death.

Age	Segment	Lower Endpoint	Upper Endpoint	APC	Lower CI	Upper CI	Test Statistic (t)	*Prob >/t/*
**Total**	1	2000	2002	41.5*	25.4	59.6	6.1	*< 0.001*
	2	2002	2019	0.3*	0	0.6	2.1	*0.049*
**00 years**	1	2000	2002	40.6	-12.8	126.7	1.5	*0.149*
	2	2002	2019	-2.4*	-3.8	-1	-3.6	*0.003*
**01-04 years**	1	2000	2019	-1.5	-3.4	0.5	-1.6	*0.127*
**05-09 years**	1	2000	2002	118.0*	24.8	281	3	*0.009*
	2	2002	2019	-1.4*	-2.5	-0.4	-2.9	*0.011*
**10-14 years**	1	2000	2002	95.8*	8.2	254.4	2.5	*0.030*
	2	2002	2014	1	-1.1	3.1	1	*0.329*
	3	2014	2019	-7.6*	-14.7	0	-2.2	*0.050*
**15-19 years**	1	2000	2002	57.1*	13.2	118.1	2.9	*0.010*
	2	2002	2019	-0.3	-1.1	0.5	-0.9	*0.373*
**20-24 years**	1	2000	2002	62.3*	25.6	109.7	4.1	*0.001*
	2	2002	2005	7.8	-7.8	26	1	*0.316*
	3	2005	2019	-2.5*	-3.2	-1.7	-7.2	*< 0.001*
**25-29 years**	1	2000	2002	59.5*	22.7	107.3	3.9	*0.002*
	2	2002	2009	3.4*	0.5	6.3	2.6	*0.024*
	3	2009	2019	-2.9*	-4.1	-1.7	-5.2	*< 0.001*
**30-34 years**	1	2000	2002	78.1*	40.2	126.3	5.1	*< 0.001*
	2	2002	2019	-0.1	-0.6	0.4	-0.4	*0.661*
**35-39 years**	1	2000	2002	59.6*	31.2	94.2	5.1	*< 0.001*
	2	2002	2019	0	-0.5	0.5	0	*0.971*
**40-44 years**	1	2000	2002	57.7*	35.7	83.2	6.5	*< 0.001*

	2	2002	2019	-1.2*	-1.5	-0.8	-6.3	*< 0.001*
**45-49 years**	1	2000	2002	55.1*	40.4	71.3	9.6	*< 0.001*
	2	2002	2010	-0.4	-1.3	0.5	-0.9	*0.365*
	3	2010	2019	-3.0*	-3.6	-2.3	-9.9	*< 0.001*
**50-54 years**	1	2000	2002	50.4*	35.6	66.7	8.6	*< 0.001*
	2	2002	2012	0	-0.7	0.6	-0.1	*0.929*
	3	2012	2019	-3.9*	-4.8	-2.9	-8.9	*< 0.001*
**55-59 years**	1	2000	2002	42.9*	29.1	58.1	7.7	*< 0.001*
	2	2002	2015	-0.3	-0.7	0	-1.9	*0.078*
	3	2015	2019	-4.9*	-6.8	-2.9	-5.3	*< 0.001*
**60-64 years**	1	2000	2002	43.4*	28.6	59.9	7	*< 0.001*
	2	2002	2019	-1.4*	-1.7	-1.2	-12.2	*< 0.001*
**65-69 years**	1	2000	2002	41.9*	23.2	63.3	5.3	*< 0.001*
	2	2002	2019	-1.6*	-2	-1.3	-10.2	*< 0.001*
**70-74 years**	1	2000	2002	39.7*	22	60	5.2	*< 0.001*
	2	2002	2019	-1.7*	-2	-1.3	-10	*< 0.001*
**75-79 years**	1	2000	2002	33.2*	20.4	47.4	6.2	*< 0.001*
	2	2002	2008	0.6	-1.2	2.4	0.7	*0.472*
	3	2008	2019	-2.1*	-2.6	-1.6	-8.6	*< 0.001*
**80-84 years**	1	2000	2002	27.5*	23	32.1	15.5	*< 0.001*
	2	2002	2006	2.0*	0.6	3.4	3.3	*0.009*
	3	2006	2017	-0.9*	-1.1	-0.7	-9.9	*< 0.001*
	4	2017	2019	-6.3*	-8.8	-3.7	-5.4	*< 0.001*
**85+ years**	1	2000	2002	26.4*	11.2	43.6	4	*0.002*
	2	2002	2007	1.5	-1.6	4.7	1.1	*0.309*
	3	2007	2019	-0.9*	-1.4	-0.4	-3.8	*0.002*

* Indicates that the Annual Percent Change (APC) is significantly different from zero at the alpha = 0.05 level.

Supplementary Table 6. Annual percent change (APC) of age-adjusted mortality rates for non-cancer caused death.

Age	Segment	Lower Endpoint	Upper Endpoint	APC	Lower CI	Upper CI	Test Statistic (t)	*Prob >/t/*
**Total**	1	2000	2002	51.2*	37.4	66.4	9.4	*< 0.001*
	2	2002	2008	11.0*	9.6	12.5	17.5	*< 0.001*
	3	2008	2019	4.8*	4.5	5.1	36.8	*< 0.001*
**00 years**	1	2000	2019	-1.3	-4.2	1.6	-1	*0.346*
**01-04 years**	1	2000	2019	-2.1	-4.9	0.8	-1.5	*0.149*
**05-09 years**	1	2000	2005	27.4*	0.6	61.5	2.2	*0.045*
	2	2005	2019	-0.9	-4.2	2.5	-0.5	*0.592*
**10-14 years**	1	2000	2019	3.2*	0.1	6.3	2.2	*0.045*
**15-19 years**	1	2000	2019	1.8	-0.5	4.2	1.7	*0.115*
**20-24 years**	1	2000	2009	8.9*	3.1	14.9	3.3	*0.005*
	2	2009	2019	-2.7	-6.2	0.9	-1.6	*0.126*
**25-29 years**	1	2000	2019	2.5*	0.8	4.2	3.1	*0.007*
**30-34 years**	1	2000	2004	13.8*	0.3	29.2	2.2	*0.046*
	2	2004	2019	0.8	-0.5	2.2	1.3	*0.225*
**35-39 years**	1	2000	2003	21.3*	3.5	42.2	2.7	*0.021*
	2	2003	2013	-2.3	-4.8	0.3	-2	*0.075*
	3	2013	2019	6.7*	2	11.6	3.2	*0.008*
**40-44 years**	1	2000	2002	36.9*	5.2	78.1	2.5	*0.023*
	2	2002	2019	0.6	-0.1	1.3	1.7	*0.101*
**45-49 years**	1	2000	2003	31.3*	11	55.4	3.5	*0.004*
	2	2003	2019	0.7	-0.2	1.6	1.6	*0.140*
**50-54 years**	1	2000	2002	48.3*	10.6	98.7	2.9	*0.013*
	2	2002	2006	7.8	-2	18.5	1.7	*0.110*

	3	2006	2019	1.3*	0.4	2.1	3.3	*0.006*
**55-59 years**	1	2000	2002	38.9*	4.5	84.8	2.5	*0.027*
	2	2002	2008	7.5*	3.4	11.7	4	*0.002*
	3	2008	2019	1.9*	1	2.8	4.6	*0.001*
**60-64 years**	1	2000	2003	31.1*	15	49.4	4.4	*< 0.001*
	2	2003	2019	3.0*	2.4	3.5	11.8	*< 0.001*
**65-69 years**	1	2000	2002	43.0*	21	69.1	4.7	*0.001*
	2	2002	2006	8.7*	2.7	15	3.2	*0.008*
	3	2006	2019	2.2*	1.7	2.6	11	*< 0.001*
**70-74 years**	1	2000	2002	50.8*	33	70.8	7.2	*< 0.001*
	2	2002	2008	7.6*	5.7	9.6	8.8	*< 0.001*
	3	2008	2019	1.7*	1.3	2.1	9	*< 0.001*
**75-79 years**	1	2000	2002	51.6*	38.7	65.8	10.6	*< 0.001*
	2	2002	2005	13.7*	7.2	20.5	5	*0.001*
	3	2005	2010	5.4*	3.7	7	7.6	*< 0.001*
	4	2010	2019	2.1*	1.7	2.5	12.7	*< 0.001*
**80-84 years**	1	2000	2002	47.9*	35.9	61	10.4	*< 0.001*
	2	2002	2005	14.9*	9	21.2	5.9	*< 0.001*
	3	2005	2010	7.8*	6.4	9.3	12.5	*< 0.001*
	4	2010	2019	2.5*	2.2	2.9	17.6	*< 0.001*
**85+ years**	1	2000	2002	52.7*	36.2	71.3	8.4	*< 0.001*
	2	2002	2006	16.0*	12.2	20	10	*< 0.001*
	3	2006	2012	7.3*	6.2	8.5	15.3	*< 0.001*
	4	2012	2019	3.5*	3	4	16.4	*< 0.001*

* Indicates that the Annual Percent Change (APC) is significantly different from zero at the alpha = 0.05 level.

Supplementary Figure 1. The proportions of primary tumors in the total cohort among different age groups.

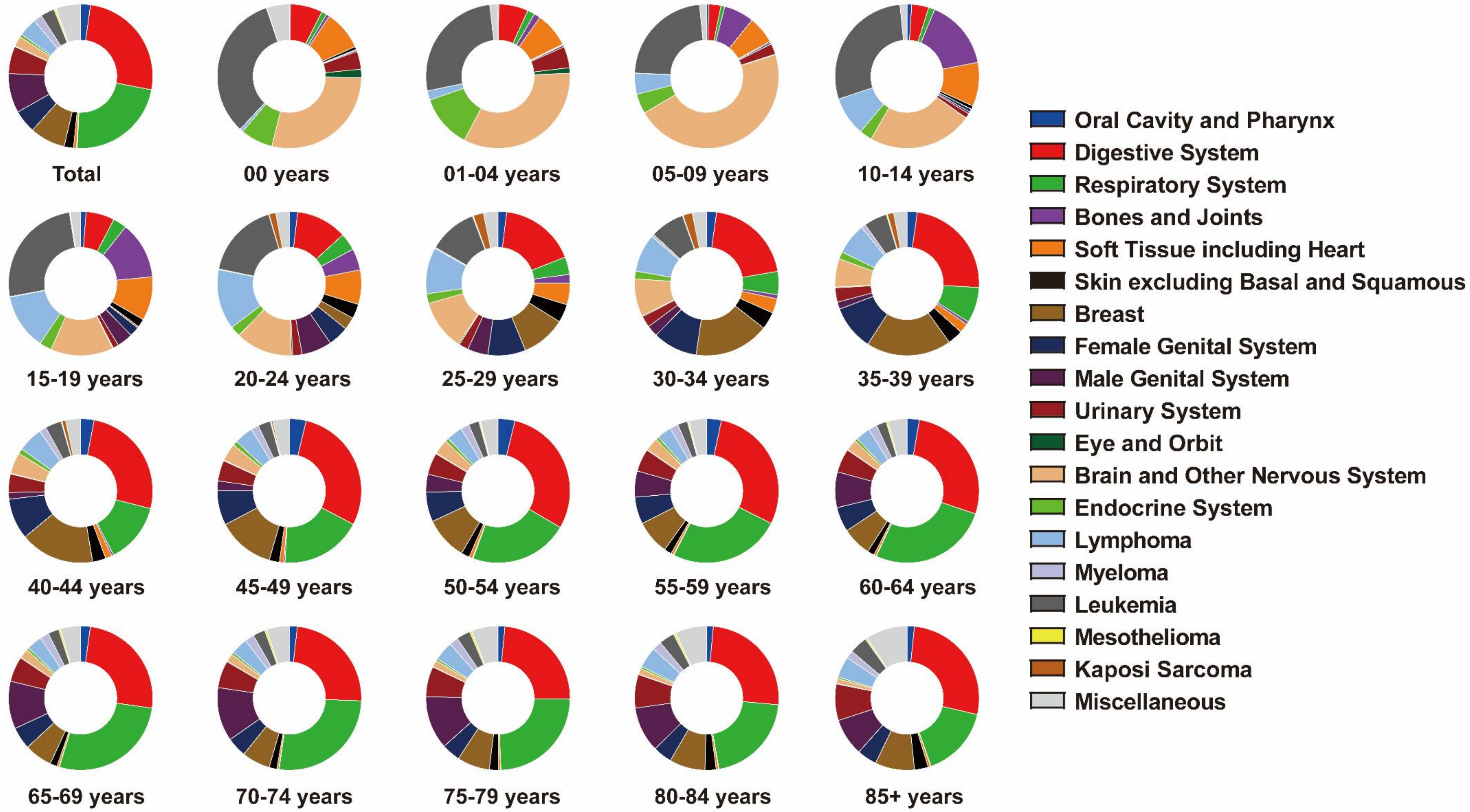

Supplementary Figure 2. The proportions of primary tumors in male patients among different age groups.

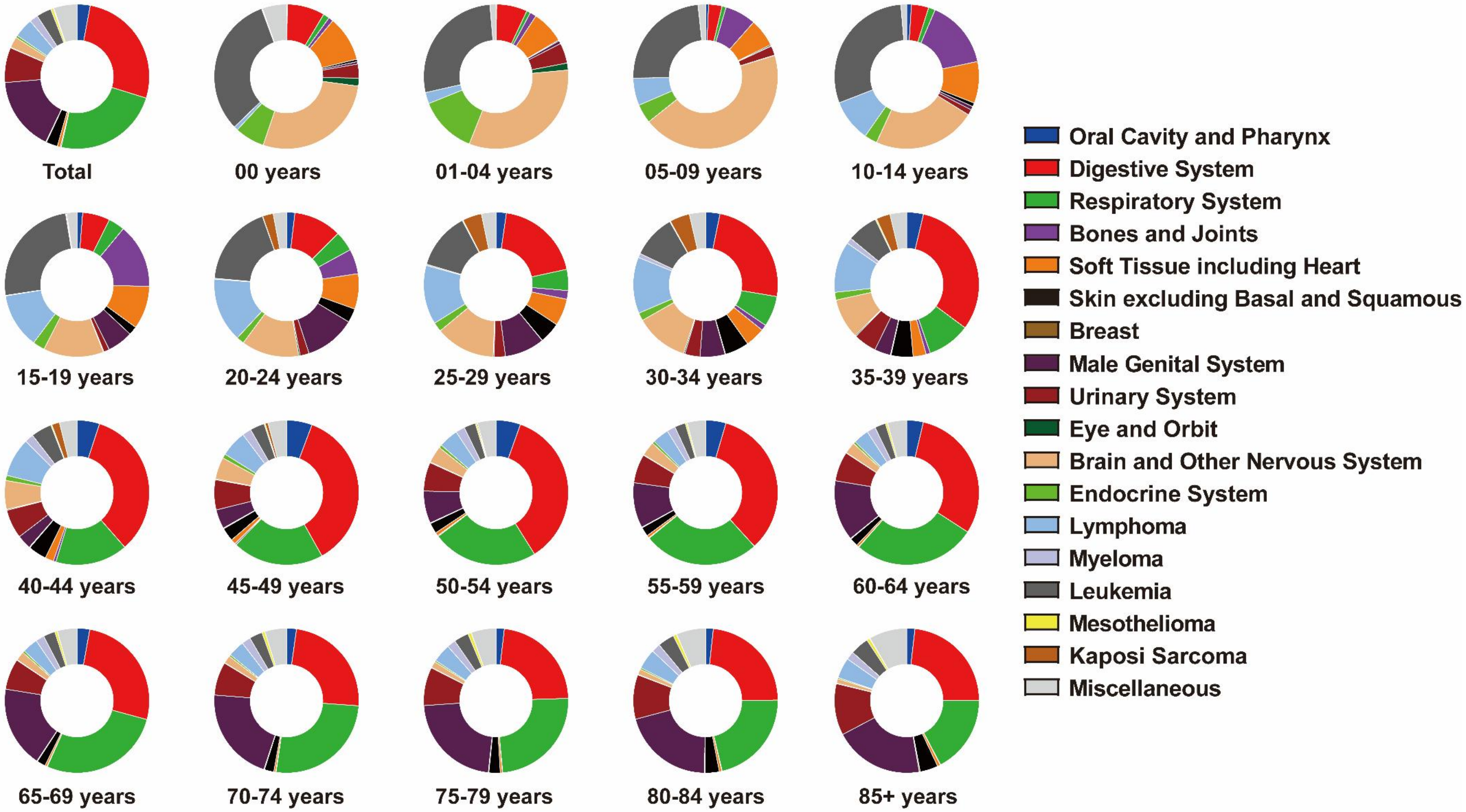

Supplementary Figure 3. The proportions of primary tumors in female patients among different age groups.

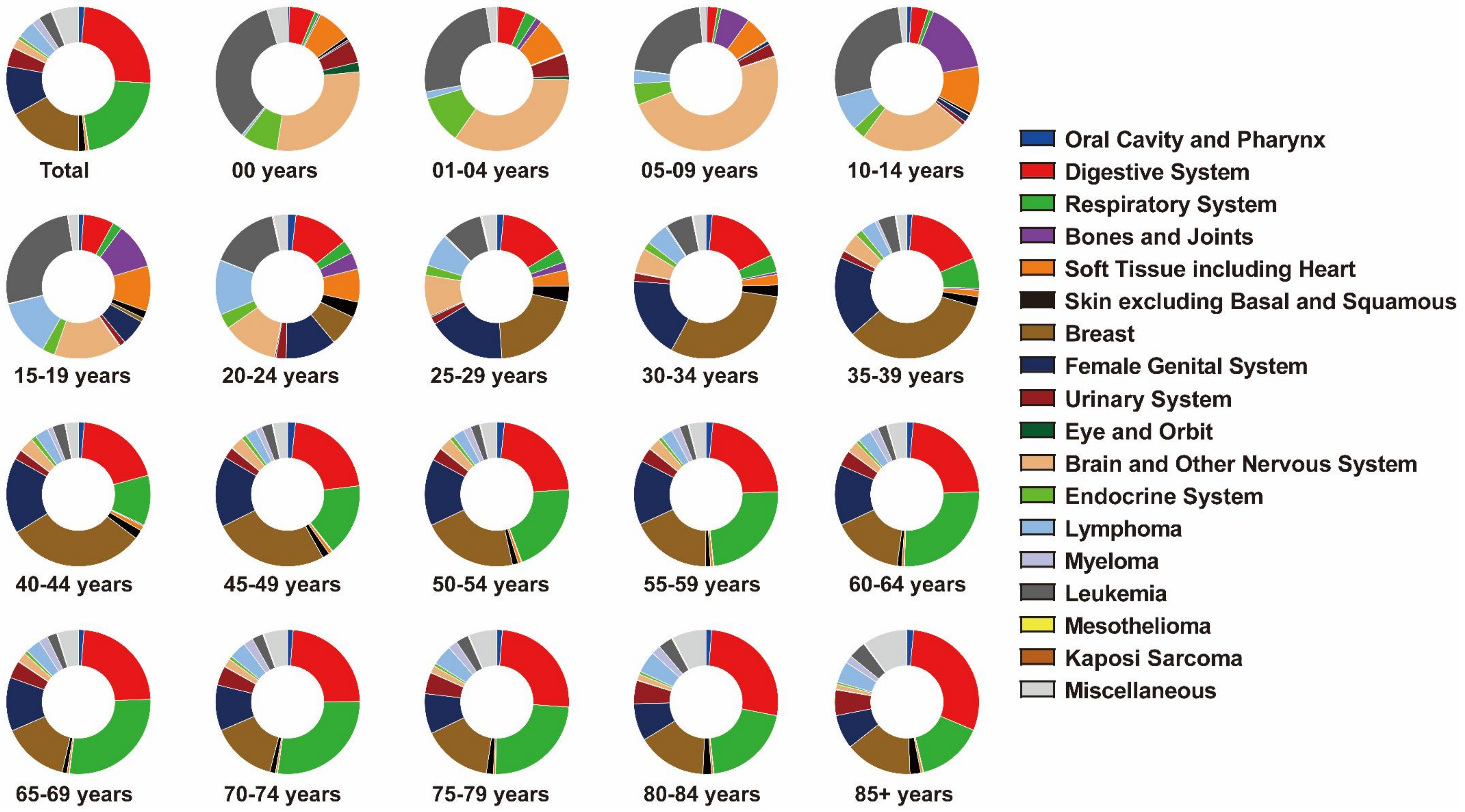

Supplement: Supplementary file 1 — Supplementary figures and tables 1, 2, 4, 5, 6. [file jcav15p1613s1.pdf]
